# Protocol: methodology for chromatin immunoprecipitation (ChIP) in *Chlamydomonas reinhardtii*

**DOI:** 10.1186/1746-4811-7-35

**Published:** 2011-11-03

**Authors:** Daniela Strenkert, Stefan Schmollinger, Michael Schroda

**Affiliations:** 1 Max-Planck-Institut für Molekulare Pflanzenphysiologie, Am Mühlenberg 1, D-14476 Potsdam-Golm, Germany

**Keywords:** Chromatin immunoprecipitation, *Chlamydomonas reinhardtii*, heat shock, formaldehyde crosslinking, real-time PCR, nucleosome occupancy, histone modification, *HSP70A*, *RBCS2*, *CYC6*

## Abstract

We report on a detailed chromatin immunoprecipitation (ChIP) protocol for the unicellular green alga *Chlamydomonas reinhardtii*. The protocol is suitable for the analysis of nucleosome occupancy, histone modifications and transcription factor binding sites at the level of mononucleosomes for targeted and genome-wide studies. We describe the optimization of conditions for crosslinking, chromatin fragmentation and antibody titer determination and provide recommendations and an example for the normalization of ChIP results as determined by real-time PCR.

## Background

Since several decades the unicellular green alga *Chlamydomonas reinhardtii *serves as a model organism for studying various aspects of cell biology [1]. However, although all three genetic compartments have been sequenced and are amenable for genetic manipulation [2], transgenic approaches frequently suffer from low transgene expression levels and from transgene silencing [3]. Recent work has shown that this is largely due to epigenetic mechanisms that frequently involve histone modifications. Several factors mediating histone modifications have already been identified in *Chlamydomonas*, mainly by Cerutti and coworkers: one of them is MUT11, a WD40-repeat protein homologous to human WDR5. Deletion of *MUT11 *resulted in the activation of single-copy transgenes and of dispersed transposons [4]. MUT11 was shown to interact with SET domain histone methyltransferases and suppression of *SET1 *by RNAi came along with a reduction in levels of monomethylated H3K4, an epigenetic mark associated with transcriptionally repressed loci [5]. Another factor is the SU(VAR)3-9-related protein SET3p. RNAi-mediated suppression of *SET3 *released the transcriptional silencing of tandemly repeated transgenes and correlated with a partial loss of monomethyl H3K9 at such loci [6]. Again another factor is the MUT9p kinase which phosphorylates H3T3 and histone H2A and is required for long-term, heritable gene silencing [7]. Furthermore, the *Chlamydomonas *enhancer of zeste homolog (EZH) catalyzes H3K27 methylation. RNAi-mediated suppression of *EZH *in *Chlamydomonas *resulted in a global increase in levels of histone H3K4 trimethylation and H4 acetylation, two characteristic marks for active chromatin, thereby leading to the release of retrotransposons and of silenced, tandemly repeated transgenes [8]. Finally, Yamasaki and coworkers found that silencing of a transgenic *RBCS2 *promoter, driving the expression of an inverted repeat construct, was associated with low levels of histone H3 acetylation and high levels of monomethylated H3K9 at the transgenic promoter [9]. Deletion of the *Elongin C *gene, which is a component of some E3 ubiquitin ligase complexes, released silencing of the transgenic *RBCS2 *promoter. The activated promoter was characterized by high levels of H3 acetylation and low levels of H3K9 monomethylation [10]. These data clearly show that *Chlamydomonas *is an excellent model organism for studying the epigenetic mechanisms underlying gene silencing.

In the past *Chlamydomonas *also served as a valuable model organism for identifying key transcription factors that regulate responses to various stress conditions, like the copper response regulator (CRR1) [11], the regulator of the phosphorus starvation response (PSR1) [12], the regulator of the carbon concentrating mechanism (CCM1/CIA5) [13,14] or the regulator of the heat shock response (HSF1) [15]. How these transcription factors regulate the expression of their target genes at the level of chromatin structure is poorly understood and only recently has become subject of investigation in our laboratory [16]. Our results revealed that CRR1 and HSF1 regulate the expression of their target genes via histone acetylation, histone methylation, nucleosome eviction and polymerase loading/activation. At each target promoter these means are employed individually to establish a characteristic chromatin state allowing for a fine-tuning of gene expression [16].

The ideal tool to study the mechanisms underlying transgene silencing and gene expression at the level of chromatin structure is chromatin immunoprecipitation (ChIP), a method that was first introduced for Drosophila [17] and has since then enormously gained in popularity. The first step in the ChIP protocol is the "freezing" of the chromatin state by infiltrating cells with formaldehyde to crosslink DNA-binding proteins with DNA. After mechanical shearing of the DNA the protein of interest is immunoprecipitated with specific antibodies together with the DNA it is binding to. The crosslinks can be reversed and the precipitated DNA is subjected to PCR for the analysis of specific loci, or to microarray hybridization/deep sequencing for the genome-wide analysis of binding sites of the protein of interest. The latter techniques have allowed for the establishment of genome-wide maps of histone modifications [18] and transcription factor binding sites [19].

Detailed ChIP protocols are published for *Tetrahymena thermophila *[20], *Drosophila *[21], yeast [22], mammalian cell lines [23], and more recently also for higher plants like *Arabidopsis *[24], maize [25] and tomato [26]. Although ChIP has been applied to *Chlamydomonas *previously [5-7,9,10,16,27], a thorough documentation of important parameters and a step-by-step protocol are yet missing, which to provide is the goal of this work.

## Materials and methods

### *Chlamydomonas *strains and cultivation conditions

*Chlamydomonas reinhardtii *strains were grown mixotrophically in Tris-acetate-phosphate (TAP) medium [1] on a rotary shaker at 24°C and ~30 μE m^-2 ^sec^-1^. Used were cell wall deficient strains CF185 [28] and 325-412 (*arg7^- ^*strain 325 transformed with pCB412 containing the wild-type *ARG7 *gene [16]) and cell walled strain CRR16 (*crr1^-^*/*arg7^- ^*strain CC3960 co-transformed with plasmids pARG7.8 and pCRR1F1B6 [11]). For heat shock experiments, cells were pelleted by a 4-min centrifugation at 24°C and 2704 × g, resuspended in TAP medium prewarmed to 40°C and incubated under agitation in a water bath at 40°C. Prior to harvest, ice was added to the cells.

### DNA fragmentation

A BANDELIN Sonopuls Sonifier HD 2070 with sonication tip MS 73 was used to shear DNA by sonication.

### Antibodies

Antibodies against the unmodified C-terminus of histone H3 were purchased from Abcam (ab1791). Antibodies against diacetyl H3K9/K14 and tetra-acetyl H4K5/K8/K12/K16 were from Upstate (06-599 and 06-866, respectively). Antibodies against VIPP2 (mock control) were affinity-purified as described previously [29].

### Buffers

#### KH buffer

20 mM Hepes-KOH pH 7.6

80 mM KCl

#### Crosslinking solution

20 mM Hepes-KOH pH 7.6

80 mM KCl

0.35% formaldehyde (Roth, ROTIPURAN p.a., 4979.1)

(as formaldehyde decays easily it is important to use fresh stocks; also always prepare the crosslinking solution freshly)

#### Lysis buffer

1% SDS

10 mM EDTA

50 mM Tris-HCl (pH 8.0)

0.25 × protease inhibitor cocktail (Roche, complete, EDTA-free, 11873580001)

#### Washing buffer 1 (low salt)

150 mM NaCl

0.1% SDS

1% Triton X-100

2 mM EDTA (pH 8.0)

#### Washing buffer 2 (high salt)

500 mM NaCl

0.1% SDS

1% Triton X-100

2 mM EDTA (pH 8.0)

#### Washing buffer 3 (LiCl)

250 mM LiCl

1% Nonidet P40 (Roche, 1754599)

1% Na-deoxycholate (Fluka, > 98.0%, 30970)

1 mM EDTA (pH 8.0)

10 mM Tris-HCl (pH 8.0)

#### TE buffer

10 mM Tris-HCl (pH 8.0)

1 mM EDTA (pH 8.0)

#### ChIP buffer

1.1% Triton X-100

1.2 mM EDTA

167 mM NaCl

16.7 mM Tris-HCl (pH 8.0)

#### Lysis buffer

1% SDS

10 mM EDTA

50 mM Tris-HCl (pH 8.0)

#### Elution buffer

1% SDS

M NaHCO_3_

(prepare freshly)

#### Stock solutions

**λDNA_soni_**: 100 μg/ml (Fermentas, #SD0011, in ChIP-buffer) [sonicated 4 times 10 sec, output control: 55%; duty cycle: 60%]

**BSA**: 10 mg/ml (Sigma, A4503, in λDNA_soni_)

**Glycogen**: 2.5 μg/μl (Roth, HP51.2, in ChIP-buffer)

**Glycine**: 1 M

**Phenol/chloroform/isoamylalcohol **(25:24:1)

**Chloroform/isoamylalcohol **(24:1)

**TE buffer **containing 20 μg/μl boiled **RNase A**

**Proteinase K: **10 mg/ml (Roth, lyophil. > 30 U/mg, 7523.3)

**Na-acetate: **3 M (pH 5.2, adjusted with acetic acid)

**NaCl: **5 M

**2x DNA loading dye: **15% Ficoll-400

**EDTA: **5 M (pH 8.0)

**Tris-HCl: **1 M (pH 8.0)

### Protocol

#### Cell harvest and crosslinking

1. Grow *Chlamydomonas *cells in 400 ml TAP medium to a density of 4-8 × 10^6 ^cells/ml.

2. Harvest 10^9 ^cells in GSA tubes by centrifuging for 3 min at 3220 × g and 24°C [4°C if cells are not subjected to further treatments].

3. [For heat shock, resuspend cells in 45 ml TAP medium prewarmed to 40°C and incubate in a 40°C water bath. Transfer cells into a 50-ml Falcon tube containing crushed ice and centrifuge for 3 min at 3220 × g and 4°C].

4. Discard supernatant and resuspend cells.

5. Add 10 ml of Crosslinking solution, mix gently for 10 min at 24°C (formaldehyde crosslinking).

6. Add 1.25 ml of 1 M glycine, agitate for 5 min at 24°C (quenching).

7. Centrifuge for 2 min at 3220 × g and 4°C, discard supernatant.

8. Resuspend cells in 1 ml KH buffer and transfer them into a 2-ml microcentrifuge tube.

9. Centrifuge for 2 min at 16, 100 × g and 4°C, discard supernatant.

10. Add 400 μl Lysis buffer; after this step cells can be stored at -80°C for several months.

#### Cell lysis and fragmentation of DNA

[In case strains with cell wall are used it is essential to add an additional 500 μl of Lysis buffer. Vortex briefly and transfer cell lysate to Ultra-Clear Centrifuge Tubes (1/2 × 2 in. (13 × 51 mm), Beckman). Cell wall-deficient strains can be further processed in 2-ml microcentrifuge tubes].

11. Sonicate on ice with 55% output control, 60% duty cycle. To achieve an average DNA fragment size of ~200 bp, sonicate 20 times 20 sec with breaks of 20 sec between each sonication cycle (avoid frothing!). The sonicator tip needs to be as close as possible to the bottom of the tube.

#### Aliquot samples

12. Transfer sonicated cell lysates to 15-ml Falcon tubes, add Lysis buffer to a total volume of 5 ml and mix carefully.

13. Centrifuge for 2 min at 3220 × g and 4°C to remove cell debris.

14. Aliquot 50 × 100 μl (100- μl aliquots correspond to chromatin from ~2 × 10^7^cells). The input chromatin can be stored for several months at -80°C, but avoid multiple freeze/thaw cycles.

*(In contrast to long-term storage, repeated freeze-thawing cycles lead to protein degradation *[30]).

#### Quality control of sheared DNA

15. Take one 100- μl aliquot and add 400 μl of Lysis buffer and 50 μl of 5 M NaCl.

16. Incubate over night at 65°C to reverse crosslinks.

17. Extract twice with 500 μl phenol/chloroform/isoamylalcohol.

18. Extract once with 500 μl chloroform/isoamylalcohol.

19. Precipitate nucleic acids by adding 55 μl of 3 M Na-acetate (pH 5.2), 1 ml of 100% EtOH and incubating for 3 h at -20°C.

20. Centrifuge for 15 min at 16, 100 × g and 4°C.

21. Wash pellet with 800 μl of 70% EtOH (take care not to remove the tiny pellet).

22. Centrifuge for 10 min at 16, 100 × g and 4°C.

23. Dry pellet and resuspend it in 10 μl TE buffer containing 20 μg/μl RNase A.

24. Incubate for 1 h at 37°C.

25. Add 10 μl of 2 × DNA loading dye and separate sheared DNA on a 1.5% agarose gel. DNA fragments should have sizes of ~200 bp.

#### Preparation of Protein A-Sepharose beads

26. Weigh-in 50 mg of Protein-A-Sepharose beads (Sigma, P3391-1G), resuspend beads in 1 ml ChIP-buffer and incubate them for 30 min at 4°C.

27. Wash beads two times with 500 μl ChIP buffer

28. Discard supernatant and add 250 μl ChIP-buffer and 250 μl λDNA_soni _(gives ~750 μl suspension with swollen beads).

#### Chromatin-Immunoprecipitation

29. Prepare antibody solutions (for example, mix 5 μl of anti-H3 antibodies with 10 μl BSA in λDNA_soni _solution and incubate for at least 30 min on ice).

30. For each antibody employed, thaw one 100- μl aliquot of chromatin solution on ice (for each strain/condition we also recommend a control without antibody).

31. Add 900 μl of ChIP buffer to each aliquot (from this step on consequently use stuffed tips to avoid DNA contaminations!).

32. Centrifuge for 20 sec at 16, 100 × g and 4°C, and transfer supernatant to microcentrifuge tubes containing the prepared antibody solutions.

33. Mix on a rotation wheel for 1 h at 4°C.

34. Centrifuge for 20 sec at 16, 100 × g and 4°C, and transfer each supernatant to microcentrifuge tubes containing 60 μl sepharose beads.

35. Mix on a rotation wheel for 2 h at 4°C.

36. Centrifuge for 20 sec at 16, 100 × g and 4°C.

37. Discard supernatant.

38. Wash the beads once with 1 ml each of Washing buffer 1, 2 and 3, and twice with 1 ml TE buffer (use washing solutions that are stored on ice and centrifuge at 4°C).

#### Elution

39. Elute precipitated chromatin with 250 μl Elution buffer for 15 min at 65°C.

40. Repeat elution with another 250 μl Elution buffer and pool eluates.

#### Reversion of the crosslink

41. Add 50 μl of 5 M NaCl and incubate overnight at 65°C.

42. To remove proteins in the precipitates add 10 μl 0.5 M EDTA (pH 8.0), 20 μl 1 M Tris-HCl (pH 8.0) and 2.1 μl proteinase K (10 mg/ml) and incubate for 1 h at 55°C.

#### DNA extraction

43. Extract once with 500 μl phenol/chloroform/isoamylalcohol.

44. Extract once with 500 μl chloroform/isoamylalcohol.

45. Precipitate DNA by adding 50 μl 3 M Na-acetate (pH 5.2), 2.5 μl glycogen (2.5 μg/μl) and 1 ml 100% EtOH and incubating for 3 h at -20°C.

46. Centrifuge for 20 min at 16, 100 × g and 4°C.

47. Wash DNA pellet with 500 μl 70% EtOH (take care not to remove the tiny pellet).

48. Centrifuge for 15 min at 16, 100 g and 4°C.

49. Dry DNA pellet in a clean bench, resuspend in 200 μl TE (pH 8.0).

50. Use 5 μl (1:40) of the precipitate for qPCR analysis (if each analysis is done with 3 technical replicates the precipitate is sufficient for 13 targets).

#### Endpoint PCR

Endpoint PCRs were carried out in 50- μl reactions containing 1 × Taq buffer (Fermentas), 5 μl chromatin immunoprecipitate, 1.5 mM MgCl_2_, 1 M betaine, 200 μM dNTPs, 2.5 U Taq polymerase (NEB), and 0.6 μM each of forward and reverse primers (see Table 1). The reaction conditions were as follows: 95°C for 5 min, followed by 27 cycles of 94°C for 1 min, 68°C for 1 min and 74°C for 30 sec. An extension step at 74°C for 5 min was added. *(Note that endpoint PCR is not an advisable method for the analysis of ChIP results as the cycle number needs to be tested empirically with dilution series until product signals are in the linear range)*.

**Table 1:** Primers used in this study.

**Nr**.	Target	Primer sequences (5' to 3')	Amplicon size (bp)
**1**	*HSP70A *promoter (endpoint PCR)	For: GACGGTGGGGAGCTCGCTGAGGC Rev: GGTGCCCAGGTCAATACCGATAGC	327

**2**	*RBCS2 *promoter (endpoint PCR)	For: GCCAGAAGGAGCGCAGCCAAACCA Rev: ACGGAGGACTTGGCAATGACGG	237

**3**	*HSP70A *promoter (qPCR)	For: CGGTATAAAAGCCCGCGAC Rev: GTGCCCAGGTCAATACCGATAG	163

**4**	*RBCS2 *promoter (qPCR)	For: CAATGCAAGCAGTTCGCATG Rev: ACGGAGGACTTGGCAATGAC	138

**5**	*CYC6 *promoter (qPCR)	For: ACACGCCCCTCATTCACAGA Rev: GCACACGAGACACTCCGAGC	121

#### qPCR

qPCR was performed using the StepOnePlus System (Applied Biosystems) and the Maxima SYBR Green kit from Fermentas. Each reaction contained the vendor's master mix, 200 nM of each primer (see Table 1), and 5 μl chromatin precipitate. The reaction conditions were as follows: 95°C for 10 min, followed by 40 cycles of 95°C for 15 sec and 65°C for 60 sec. For each target we made sure that melting curves had single peaks and that only one PCR product was visible on 1.5% agarose gels. Non-template controls were generally included.

### Comments

#### Crosslinking and reversal

As pointed out previously, the conditions used for the crosslinking of DNA with proteins are crucial for ChIP analyses and need to be optimized for each individual organism [21,23,25]. Important parameters are the amount of input material, the formaldehyde concentration and the crosslinking time. Protocols for higher plants start out from 1-5 g of plant tissue and crosslinking is done for 10-15 min with 1-3% formaldehyde [24-26,31]. Protocols for *Chlamydomonas *start out from 2-4 × 10^7 ^cells and crosslinking is done for 5-10 min with 0.75-1% formaldehyde [5,9]. Following crosslinking, chromatin is sheared by sonication to average fragment sizes ranging between 200 and 3000 bp (higher plant protocols) [24-26] and 500-1000 bp (*Chlamydomonas *protocols) [5,6,9].

**Figure 1:**
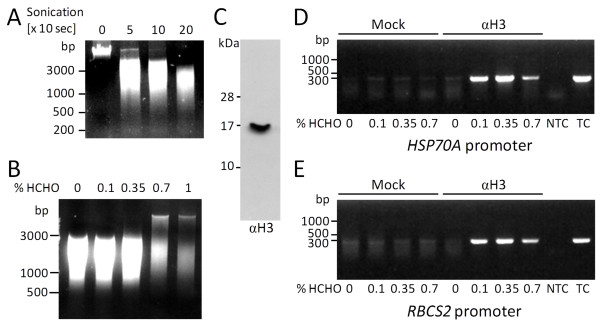
**Optimization of sonication conditions to yield ~2000-bp chromatin fragments and of formaldehyde concentrations for efficient crosslinking**. **A: **2 × 10^7 ^cells of *Chlamydomonas *strain CF185 were sonicated 0, 5, 10 and 20 times for 10 seconds. DNA was extracted with phenol/chloroform/isoamylalcohol, separated on a 1.5% agarose gel and stained with Ethidium bromide. **B: **2 × 10^7 ^cells of strain CF185 were incubated for 10 min with formaldehyde (HCHO) at the concentrations indicated. After sonication of cells crosslinks were reversed by over-night incubation at 65°C. DNA was extracted and visualized as in (A). **C**: whole-cell proteins corresponding to 2 μg chlorophyll from strain cw15-325 were separated on a 15% SDS-polyacrylamide gel and analyzed by immunoblotting using antibodies against histone H3. **D, E: **ChIP was done with preimmune serum (mock) and with antibodies against histone H3 using chromatin as input that was crosslinked with formaldehyde at the indicated concentrations. Precipitated DNA was amplified using primers targeting the *HSP70A *promoter (D) or the *RBCS2 *promoter (E) and visualized as in (A). NTC-non-template control; TC-template control based on 10% input DNA.

To optimize crosslinking conditions for *Chlamydomonas*, we used as fixed parameters 2 × 10^7 ^cells as starting material, a crosslinking time of 10 min, and a DNA fragment size of 1000-3000 bp (20 sonication pulses of 10 sec each; Figure 1A). As a variable parameter, we employed formaldehyde concentrations ranging from 0.1 to 1%. As shown in Figure 1B, DNA of high molecular weight and less DNA fragments in the 1000-3000 bp range were recovered when formaldehyde concentrations of 0.7% and higher were used. An obvious interpretation for this observation is that at formaldehyde concentrations above 0.7% chromatin is crosslinked to higher-order structures that resist fragmentation by sonication and from which crosslinks cannot be fully reversed. Decrosslinking is performed by over-night incubation at 65°C [21] and represents an essential step, as DNA-protein complexes enter the phenol phase and are removed during phenol extraction. Presumably because of the high protein content in compact, over-crosslinked structures, they tend to be removed during phenol extraction. Similar observations with over-crosslinking at too high formaldehyde concentrations or crosslinking times were also reported by others [21,23,26].

To elucidate how the formaldehyde concentration used for crosslinking affects ChIP efficiency, we used antibodies against the unmodified C-terminus of histone H3 (Figure 1C) to immunoprecipitate DNA crosslinked to histones. Subsequently, precipitated DNA fragments from promoters *HSP70A *and *RBCS2 *were amplified by endpoint PCR. As shown in Figures 1D and 1E, the largest amounts of promoter DNA were precipitated when chromatin was crosslinked with a formaldehyde concentration of 0.35%. Insufficient crosslinking at lower formaldehyde concentrations and over-crosslinking at higher concentrations both impaired ChIP efficiency.

#### Fragmentation of DNA for high-resolution ChIP

In *Chlamydomonas*, the nucleosome repeat length was determined to have values of 156 and 160 bp at the *RBCS2 *and *HSP70A *loci, respectively, whereas a value of 178 bp was detected in bulk chromatin [32]. Hence, a DNA fragment size of 1000-3000 bp, as used above for determining the optimal formaldehyde concentrations for crosslinking (Figure 1), would encompass between 6 and 19 nucleosomes. Fragments of this size are therefore neither suitable for studying nucleosome occupancy and histone modifications at high resolution, nor for precisely determining transcription factor binding sites.

**Figure 2:**
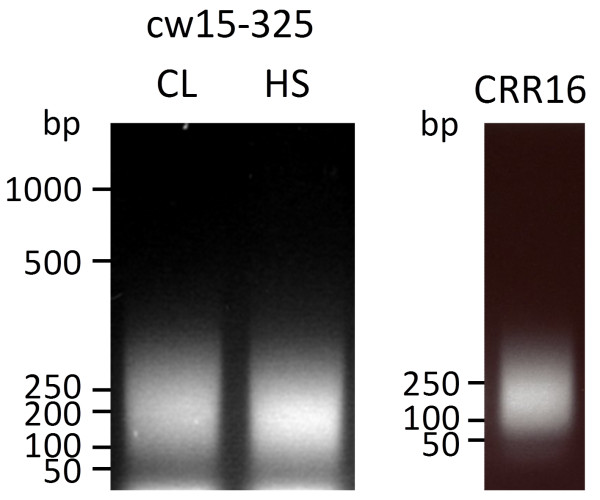
**Optimization of sonication conditions to yield ~200-bp chromatin fragments**. 2 × 10^7 ^cells of *Chlamydomonas *strains cw15-325 and CRR16 were sonicated 20 times 20 sec. DNA was extracted, separated on a 1.5% agarose gel and stained with Ethidium bromide.

To reduce the size of chromatin fragments as input for ChIP we increased the sonication time to 20 times 20 sec. This yielded DNA fragments with an average fragment size of ~200 bp, basically corresponding to mononucleosomes (Figure 2) [16]. Note that when using such small chromatin fragments, PCR amplicon sizes should not exceed 120-160 bp. Note also that for cell walled *Chlamydomonas *strains lysis and chromatin shearing need to be carried out with a larger volume of lysis buffer. This increases the effective SDS concentration to efficiently solubilize the proteinaceous cell wall.

#### Titration of antibodies

ChIP needs to be performed either under conditions where the investigated epitope is in large excess (e.g. when nucleosome occupancy or abundant histone modifications are analyzed), or where the antibodies used are in large excess (e.g. when transcription factors or rare histone modifications are investigated). In the former case there should be a linear relationship between the amount of antibodies applied and antigen precipitated. In the latter case the amount of antigen precipitated under the respective condition should be constant and independent of the amount of antibodies used. To ensure that these premises are fulfilled it is important to titrate the antibodies used. Titration is also advisable to reduce costs, as commercially available antibodies against histones and histone modifications are expensive. Moreover, antibody titers may vary from batch to batch. Ideally, if the antigen is in excess, the amount of antibodies used should be adjusted such that robust signal intensities are obtained compared to a mock control.

**Figure 3:**
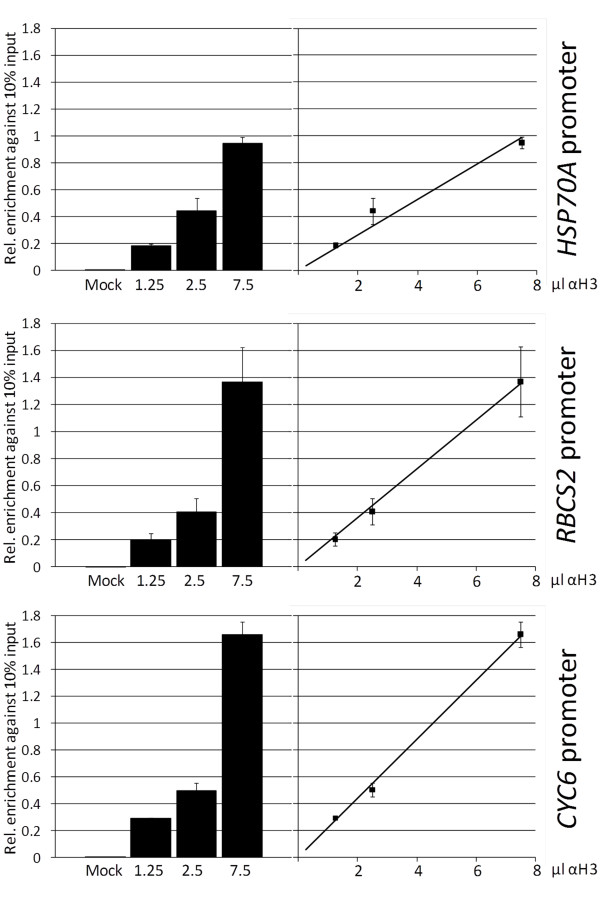
**Testing for the linearity of ChIP efficiency with antibodies against histone H3**. ChIP was done on 2 × 10^7 ^cells of *Chlamydomonas *strain CRR16 crosslinked with 0.35% formaldehyde. Cells were not subjected to stress. Chromatin was sonicated to fragments of ~200-bp and immunoprecipitated with affinity-purified antibodies against VIPP2 (mock) or with the indicated volumes of a suspension containing antibodies against histone H3. Precipitated DNA was amplified by qPCR using primers targeting promoters *RBCS2*, *HSP70A*, and *CYC6*. Shown are standard errors from 3 technical replicates. Values are normalized against those obtained with 10% input DNA.

To give an example for a proper antibody titration, we performed ChIP using different dilutions of antibodies directed against the unmodified C-terminus of histone H3 and employed affinity-purified antibodies against vesicle-inducing protein in plastids 2 (VIPP2) as mock control. The amount of precipitated chromatin from promoters *HSP70A*, *RBCS2 *and *CYC6 *relative to input DNA was quantified by real-time PCR (qPCR). As shown in Figure 3, we observed a linear relationship between the amount of antibodies used and the qPCR signal obtained for all three promoters. The smallest volume of antibodies used (1.25 μl) gave qPCR signals that were significantly stronger than those of the mock control but were still in the linear range. Hence, 1.25 μl of antibody suspension (diluted to a larger volume to reduce pipetting errors) would be more than sufficient for studying nucleosome occupancy at these promoters. The regression lines obtained suggest that nucleosome occupancy is highest at the *CYC6 *promoter, intermediate at *RBCS2 *and lowest at *HSP70A*, hence corroborating our earlier findings [16].

#### Considerations regarding data analysis

The advantages and disadvantages of different normalization approaches to handle qPCR data from ChIP experiments have been thoroughly discussed by Haring et al. [25]. We will therefore restrict ourselves to describing how we do normalization and why [16].

1. No matter whether antibodies against core histones, histone modifications or transcription factors are used for ChIP, the qPCR signals from ChIPs using specific antibodies need to be significantly stronger than those from ChIPs using mock controls. Of course, the investigated antigen must be present at the target site investigated. The choice of a proper control antibody depends on the nature of the specific antibody used. For example, if the specific antibody is affinity-purified (like that against the C-terminus of histone H3), the control antibody-targeting an unrelated antigen-should be affinity-purified as well (like that against VIPP2).

2. Determine nucleosome occupancy and/or histone modifications at the locus of interest by normalizing qPCR signals from ChIPs with antibodies against a core histone and/or specific histone modifications with the qPCR signal from input chromatin (%IP). This eliminates variations in PCR efficiency for the respective locus and makes comparisons between different loci (and different PCR runs) possible.

3. Express nucleosome occupancy and/or histone modifications determined for the locus of interest relative to that of a control locus known to contain nucleosomes and/or the histone modifications under investigation (note that when changes in nucleosome occupancy or histone modifications in response to e.g. a change in environmental conditions are monitored it is important to ensure that neither histone occupancy nor modifications are affected at the respective control locus). As values for target and control loci are obtained from the same ChIP experiment, technical variations within that experiment are eliminated. This procedure also allows integrating biological replicates where for example antibody batches with varying efficiencies were used.

4. As nucleosome occupancy may vary considerably between different loci (or even at the same locus in response to environmental cues), histone modifications generally need to be expressed relative to nucleosome occupancy.

When investigating histone modifications in *Chlamydomonas*, it is important to keep in mind that the amino acid sequences of *Chlamydomonas *histones are not entirely conserved with those of mammals, against which many commercially available antibodies for the detection of histone modifications are directed. For instance, *Chlamydomonas *histone H3 contains a threonine instead of a serine at position 28 and the alanine at position 29 found in mammalian histone H3 is deleted in the *Chlamydomonas *protein. As a consequence, commercially available antibodies against methyl-H3K27 are not functional in *Chlamydomonas *[8].

#### Heat stress leads to rapid nucleosome remodeling at the RBCS2 promoter

We have shown previously that heat stress caused reductions in levels of H3 and H4 acetylation at the *Chlamydomonas RBCS2 *promoter by ~2- and ~6-fold, respectively, which correlated with a ~20% increase in nucleosome occupancy [16]. Interestingly, this effect was not mediated by HSF1 and might be part of a global, heat shock-induced loss of histone acetylation that was first observed in *Drosophila *[33]. We could also show that HSF1-dependent remodeling of nucleosomes at the *Chlamydomonas HSP22F *promoter occurred within the first minute after onset of heat stress, right after binding of HSF1 to the promoter [16]. We wondered whether HSF1-independent remodeling of nucleosomes at the *RBCS2 *promoter during heat stress occurred as fast as HSF1-dependent remodelling at *HSP22F*. To address this question, we employed ChIP to monitor nucleosome occupancy and levels of H3 and H4 acetylation at the *RBCS2 *promoter during the first 10 min of heat stress.

For the evaluation of this experiment we followed the normalization guidelines given above: (1) qPCR signals from precipitates generated with specific antibodies were much stronger than those obtained from precipitates generated with mock antibodies (not shown). (2) qPCR signals obtained with antibodies against histone H3, di-acetylated H3 and tetra-acetylated H4 were first normalized to those gained with 10% input DNA and (3) subsequently normalized to the respective values obtained for the *CYC6 *control promoter (nucleosome occupancy and levels of H3/H4 acetylation at the *CYC6 *promoter were shown not to change during heat stress [16]). (4) qPCR signals obtained with antibodies against acetylated histones were expressed relative to those obtained with antibodies against unmodified histone H3.

**Figure 4:**
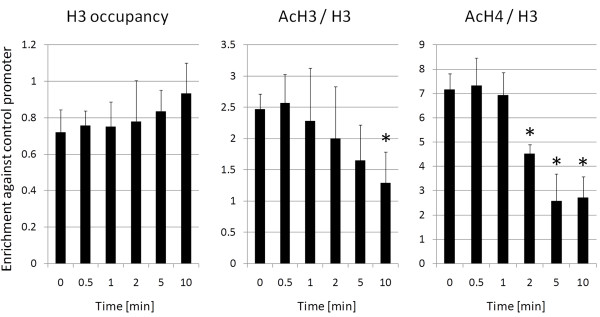
**Analysis of chromatin remodeling at the *RBCS2 *promoter within the first 10 minutes after onset of heat stress**. *Chlamydomonas *cells of strain 325-412 were subjected to heat stress and samples for ChIP were taken prior to and at the indicated time points after shifting from 24°C to 40°C. qPCR was used to quantify the amount of *RBCS2 *promoter fragments precipitated with antibodies against histone H3, di-acetylated histone H3, and tetra-acetylated histone H4. The enrichment relative to 10% input DNA was calculated. All values are normalized against those obtained for the *CYC6 *promoter (control). qPCR data from ChIPs with antibodies against modified histones are given relative to the nucleosome occupancy. Error bars indicate standard errors from two biological replicates, each analyzed in triplicate. Asterisks indicate the significance of change compared with non-stress conditions (Holm-Sidak t-test, p value ≤ 0.05).

As shown in Figure 4, a significant decrease in levels of histone H4 acetylation at the *RBCS2 *promoter was observed 2 min after onset of heat stress. Trends towards reduced levels of H3 acetylation and increased histone occupancy were also discernible after about 2 min of heat stress. Hence, HSF1-independent nucleosome remodeling at the *RBCS2 *promoter after heat stress is fast (within 2 min), but not as fast as HSF1-dependent nucleosome remodeling at the *HSP22F *promoter (within 1 min).

## Conclusions

We describe here a detailed chromatin immunoprecipitation protocol for *Chlamydomonas*. We demonstrate the optimization of the most important parameters, which are the crosslinking conditions, the shearing of chromatin, the titration of antibodies and the normalization of results obtained by qPCR. Compared to earlier ChIP protocols described for *Chlamydomonas *[5,6,9], our protocol bears the following improvements: (i) it combines cell lysis and chromatin fragmentation in a single step. (ii) It facilitates high-resolution analyses as chromatin is fragmented into mononucleosomes rather than tri- to pentanucleosomes. (iii) It allows precipitating transcription factors and associated DNA [16]. By demonstrating that after heat shock histone acetylation levels at the *RBCS2 *promoter rapidly decrease and nucleosome occupancy rapidly increases we provide an example for the suitability of our ChIP protocol for monitoring changes of chromatin structure in a minutes scale. Our protocol will prove useful for targeted and genome-wide studies of chromatin structure in *Chlamydomonas *and other eukaryotic microalgae.

## Competing interests

The authors declare that they have no competing interests.

## Authors' contributions

DS participated in the design of the study, carried out all experiments and drafted the manuscript. SS participated in the design of the study and helped with data evaluation. MS conceived and coordinated the study, and wrote the manuscript. All authors read and approved the final manuscript.
